# Robotic Cardiac Surgery: What the Young Surgeon Should Know

**DOI:** 10.21470/1678-9741-2020-0437

**Published:** 2020

**Authors:** Jef Van den Eynde, Ludovic Melly, Gianluca Torregrossa, Wouter Oosterlinck

**Affiliations:** 1 Department of Cardiovascular Diseases, Research Unit of Cardiac Surgery, University Hospitals Leuven, Leuven, Belgium.; 2 Department of Cardiac Surgery, CHU UCL Namur, Yvoir, Belgium.; 3 Department of Cardiovascular Surgery, Mount Sinai St. Luke’s Hospital, New York, NY, USA.

Since the introduction of minimally invasive cardiac surgery in 1995, robotic systems have gained popularity. The first successful cases were reported in 1998 by Carpentier^[1]^ and Mohr^[2]^, who independently performed mitral valve surgery using prototypes from the da Vinci Surgical System (Intuitive Surgical Inc., Sunnyvale, California, USA). Shortly thereafter, the first generation of devices received FDA approval and, in the years ensuing, new generations were released. Apart from avoiding sternotomy and using small portal incisions instead, robotic tele-manipulators provide three-dimensional (3D) and magnified visualization. They are also equipped with articulating instruments that have seven degrees of freedom of motion, hence significantly optimizing dexterity. Various studies have demonstrated advantages of robotic cardiac surgery, such as reduced hospital length of stay, complications, and mortality^[3,4]^. The important benefits of robotic surgery on fast recovery and quality of life are illustrated by the testimony of a patient operated on at one of the author’s institutions: https://www.linkedin.com/posts/gianluca-torregrossa-70b4965b_covid19-cardiacsurgery-robotic-ugcPost-6695354516503633920-K61f/.

On the other hand, the main controversy remains the cost. The purchase price of a robotic system can exceed US$2 million with ongoing maintenance costs of US$100,000 each year^[5]^. Depending on the calculation methods (with amortization of the initial investment) and the surgical procedure, using the robot generates an extra cost of 6-13% (US$600-4000). However, results from a study by Morgan et al.^[6]^ suggest that improvements in postoperative quality of life and a more expeditious return to work might make robotic surgery overall cost-effective. Furthermore, a reduction in cost is expected, as more companies will enter the market and start to compete.

It is the position of the authors that the potential of robotic cardiac surgery should be embraced as much as possible to maintain the medium- and long-term benefit of surgery while minimizing trauma. As robotic surgery might eventually play a bigger role in the cardiac surgery landscape, future generations of surgeons should be prepared for this scenario. We believe that efforts should be focused on three main pillars: training, research, and collaborative networks.

## Training

Robotic surgery has challenged the notorious Halstedian teaching adage of “See one, do one, teach one”. Whereas conventional open surgery required constant hands-in-the-patient cooperation from the resident, the latter is now left at the bedside to do suctioning or in a second robotic console to watch the supervising surgeon’s actions^[7]^. A potential reduction in the chances to get hands-on practice in the operating theatre does not necessarily imply inferior learning chances: with today’s technology, residents can see and study the procedure in much more detail than if it is performed via sternotomy. Furthermore, robotic cardiac surgery has a steep learning curve and experienced surgeons usually require only a couple of hundreds of procedures to become competent^[5]^. Nonetheless, exposure to robotic surgery for most surgical trainees remains very limited as dedicated training programs are scarce. A shift in training paradigms will be required to meet these needs for future surgeons.

Various novel training modalities based on simulation have been tested in the setting of robotic cardiac surgery: dry labs, wet labs (cadaveric and animal models), and virtual reality simulation. Valdis et al.^[8]^ performed the first randomized controlled trial to compare these modalities, demonstrating that all three helped surgical trainees to achieve a proficiency level within an accelerated time frame. Wet labs provided the most efficient training, although virtual reality simulations showed to be a valuable alternative in case the appropriate resources for wet labs are not available.

Although these new modalities would theoretically enable residents to train on their own anywhere at any time, it is recommended that they be guided by expert robotic surgeons. Ideally, residents should visit experienced centers to receive advanced training. Dedicated “robotic fellowships” and specialization courses are currently available in an increasing number of institutions (Table 1). Residents are encouraged to actively look out for such opportunities.

**Table 1:** Currently available robotic fellowships and specialization courses.

Name	Institution	Link
**Fellowships**		
STS/TSF Advanced Robotic Cardiac Surgery Fellowship	Society of Thoracic Surgeons The Thoracic Surgery Foundation	https://thoracicsurgeryfoundation.org/roboticcardiacfellowship/
AATS Foundation Cardiac Surgical Robotic Fellowship	AATS Foundation	https://www.aats.org/aatsimis/AATSWeb/Foundation/Programs/Programs/Cardiac_Surgical_Robotics_Program/Cardiac_Surgical_Robotics_Program.aspx
Advanced Robotic Cardiac Surgery Fellowship	Division of Cardiothoracic Surgery, Emory University School of Medicine, GA, USA	http://www.surgery.emory.edu/training/ct-surgery-residency-fellowships/advanced-robotic-cardiac-surgery-fellowship.html
Minimally Invasive CT Surgery Fellowship	Department of Cardiovascular and Thoracic Surgery, West Virginia University, WV, USA	https://medicine.hsc.wvu.edu/cardiovascular-and-thoracic-surgery/fellowships/cardiothoracic-surgery-residency/minimally-invasive-ct-surgery-fellowship/
Resident Robotic Surgery Training	Department of Cardiothoracic Surgery, Weill Cornell Medicine, NY, USA	https://ctsurgery.weillcornell.org/education/resident-robotic-surgery-training
**Training courses**		
STS Workshop on Robotic Cardiac Surgery	Society of Thoracic Surgeons Annual event	https://www.sts.org/meetings/live-courses/2020-sts-workshop-robotic-cardiac-surgery-agenda
Robotic Training Courses at ORSI Academy	ORSI Academy, Belgium Throughout the year	https://www.orsi-online.com/en/training
(ICRS) Robotic Surgery course	International College of Robotic Surgeons, World Laparoscopy Hospital	https://www.laparoscopyhospital.com/roboticsurgerytraining.html

## Research

An important determinant of the future of robotic cardiac surgery will be the development of new technical innovations, such as other robotic platforms, new instruments and new devices (anastomotic connectors, knot tying tools…). An example is the addition of tactile feedback to the robotic system, which is required for various tasks such as tissue retraction, dissection, and manipulation^[9]^. As most of the learning curve is currently spent on compensating for the absence of tactile feedback via visual clues, such vibrotactile systems might make the acquisition of robotic surgical skills faster. Another path to follow could be the development of software that analyses the images obtained from the camera, or that overlays 3D echocardiography and computerized tomography (CT) scan images onto the surgical field as well as the aid of artificial intelligence to improve repeated tasks. Close collaboration between surgeons and engineers will be essential to set research priorities and ensure incorporation of new tools in clinical practice.

The relative risks and benefits of robotic *versus* nonrobotic surgery have not yet been sufficiently mapped out. Furthermore, as surgical technique evolves, continuous effort should be spent on the monitoring of outcomes. Such data will be instrumental in validating the safety and cost-effectiveness of robotic surgery. Young surgeons can play an important role in the creation and maintenance of comprehensive collective clinical databases at institutional, national, and professional society level.

## Collaborative Networks

The ultimate goal of robotic cardiac surgery is to maximize benefits while minimizing risks when offering a surgical approach to patients. This depends on a coordinated effort by multiple stakeholders. Rather than a siloed approach where every center is focused on its own progress, the combination of forces can lead to an acceleration of discovery and translation, as well as a reduction of costs.

Whenever possible, the purchase of robotic systems should be coordinated between centers or networks and arrangements should be sought to make shared use of robotic surgical systems. Besides, it should be encouraged that experienced surgeons proctor in nearby centers to train other surgeons for robotic procedures. Furthermore, collaborative networks are very helpful in establishing a robotic surgical program, as intellectual advice and practical support can be provided by and shared between centers.

It is clear that robotic approaches have the potential to bring major changes in the practice of cardiac surgery in the future. Not only will training programs have to adapt, but also important technological advances are to be expected. Furthermore, collaboration between staff of the same center and between centers will become mandatory and even vital. Keeping the three pillars in mind, the young surgeon should get prepared for this exciting future.
